# Regulation of Amphiregulin Gene Expression by β-Catenin Signaling in Human Hepatocellular Carcinoma Cells: A Novel Crosstalk between FGF19 and the EGFR System

**DOI:** 10.1371/journal.pone.0052711

**Published:** 2012-12-20

**Authors:** Maria U. Latasa, Fabiana Salis, Raquel Urtasun, Oihane Garcia-Irigoyen, Maria Elizalde, Iker Uriarte, Monica Santamaria, Francesco Feo, Rosa M. Pascale, Jesús Prieto, Carmen Berasain, Matías A. Avila

**Affiliations:** 1 Division of Hepatology and Gene Therapy, Centre for Applied Medical Research, University of Navarra, Pamplona, Spain; 2 Division of Experimental Pathology and Oncology, Department of Clinical and Experimental Medicine & Oncology, University of Sassari, Sassari, Italy; 3 Centro de Investigación Biomédica en Red en el Área temática de Enfermedades Hepáticas y Digestivas, University Clinic, University of Navarra, Pamplona, Spain; Kanazawa University, Japan

## Abstract

Hepatocellular carcinoma (HCC) is the most prevalent liver tumor and a deadly disease with limited therapeutic options. Dysregulation of cell signaling pathways is a common denominator in tumorigenesis, including hepatocarcinogenesis. The epidermal growth factor receptor (EGFR) signaling system is commonly activated in HCC, and is currently being evaluated as a therapeutic target in combination therapies. We and others have identified a central role for the EGFR ligand amphiregulin (AR) in the proliferation, survival and drug resistance of HCC cells. *AR* expression is frequently up-regulated in HCC tissues and cells through mechanisms not completely known. Here we identify the β-catenin signaling pathway as a novel mechanism leading to transcriptional activation of the *AR* gene in human HCC cells. Activation of β-catenin signaling, or expression of the T41A β-catenin active mutant, led to the induction of *AR* expression involving three specific β-catenin-Tcf responsive elements in its proximal promoter. We demonstrate that HCC cells expressing the T41A β-catenin active mutant show enhanced proliferation that is dependent in part on AR expression and EGFR signaling. We also demonstrate here a novel cross-talk of the EGFR system with fibroblast growth factor 19 (FGF19). FGF19 is a recently identified driver gene in hepatocarcinogenesis and an activator of β-catenin signaling in HCC and colon cancer cells. We show that FGF19 induced *AR* gene expression through the β-catenin pathway in human HCC cells. Importantly, AR up-regulation and EGFR signaling participated in the induction of cyclin D1 and cell proliferation elicited by FGF19. Finally, we demonstrate a positive correlation between *FGF19* and *AR* expression in human HCC tissues, therefore supporting in clinical samples our experimental observations. These findings identify the AR/EGFR system as a key mediator of FGF19 responses in HCC cells involving β-catenin signaling, and suggest that combined targeting of FGF19 and AR/EGFR may enhance therapeutic efficacy.

## Introduction

Hepatocellular carcinoma (HCC) is the most frequent form of primary liver cancer and a deadly disease [1], [2]. HCC is highly prevalent in Sub-Saharan Africa and eastern Asia, but its incidence has increased significantly in Western countries [3], [4]. In most cases HCC slowly develops on a background of chronic liver inflammation and injury mainly caused by hepatitis B virus and hepatitis C virus infections, chronic alcohol consumption and non-alcoholic steatohepatitis [1], [2], [5]. The high mortality rate in patients with HCC is mainly due to the lack of effective therapeutic options to treat the disease in intermediate or advanced stages, that is when most cases are diagnosed [6], [7]. HCC is highly resistant to treatment, including systemic chemotherapeutic agents like doxorubicin or 5-fluorouracyl, radiotherapy and immune or hormonal therapies [6]–[8]. Such resistance to treatment stems in part from the molecular complexity and heterogeneity of this type of tumor, as evidenced in different high-throughput genomic studies performed over the past decade [9]–[11]. However, these and other molecular approaches have also identified a set of signaling and gene regulatory pathways that are commonly dysregulated in HCC tissues and cells. In fact, besides frequent mutations in the tumor suppressor gene *TP53*, common denominator to most HCCs were alterations in intracellular signal transduction pathways triggered by growth factors [7], [12]–[15]. These include the hepatocyte growth factor (HGF), insulin-like growth factor (IGF), vascular endothelial growth factor (VEGF), transforming growth factor β (TGFβ), the epidermal growth factor (EGF) family of ligands, as well as the wingless (Wnt)/β-catenin and hedgehog signaling systems. The functional relevance of these alterations in hepatocarcinogenesis has been demonstrated in numerous experimental studies [12]–[15]. However, a more compelling evidence of their pathological importance was the antitumoral efficacy shown by the multitarget kinase inhibitor sorafenib in patients with HCC [7], [8]. Other molecules targeting signaling pathways, such as EGF and VEGF receptors inhibitors, are currently being tested in clinical trials, either alone or in combination with sorafenib with the aim of increasing therapeutic efficacy [7], [14]. The rationale behind the combination of targeted therapies lies in part in the extensive cross-talks between signaling pathways in HCC cells, which are increasingly recognized to limit antitumoral effects and to participate in the development of drug resistance [16].

The EGF receptor (EGFR) is perhaps one of the signaling systems that undergo most extensive cross-talk in cancer cells, including HCC cells, being considered as a “signaling hub” where different growth and survival signals converge [17]. These interactions with other signal pathways may occur at different levels, such as the induction of EGFR ligands expression and release, the physical interaction between the EGFR and other receptors (e.g. IGFR1), or the EGFR-mediated up-regulation of other growth factors (e.g. connective tissue growth factor, CTGF) [18], [19]. The EGFR is a transmembrane tyrosine kinase receptor that can be bound and activated by a family of ligands that in addition to EGF include amphiregulin (AR), transforming growth factor a (TGFα), epiregulin, and heparin-binding EGF (HB-EGF) [17]. We and others have previously demonstrated that among these EGFR ligands AR plays a key role in the growth, survival and chemoresistance of cancer cells, including HCC cells [19]–[23]. Moreover, recent reports show that EGFR activation, and in particular AR expression, may limit HCC cells’ response to sorafenib, emphasizing the importance of AR/EGFR signaling in HCC development and treatment [24], [25]. However, in spite of these relevant effects of AR the mechanisms responsible for its up-regulation in HCC tissues and cell lines have not been so far examined [20], [21]. As mentioned above, one of the signaling pathways most frequently dysregulated in HCC is the Wnt/β-catenin system [10]–[15], [26], [27]. Cross-talks between Wnt/β-catenin signaling and the EGFR system have been reported in different cell types, including liver parenchymal cells [28]–[31], therefore we decided to explore the potential regulation of AR gene expression by β-catenin signaling in human HCC cells. Here we demonstrate that AR expression can be modulated by β-catenin pathway activation. Moreover, we identify fibroblast growth factor 19 (FGF19), a soluble growth factor recently involved in hepatocarcinogenesis and a β-catenin pathway activator [32], [33], as a novel agonist for AR gene expression in human HCC cells. We also provide evidence supporting a role for AR in the growth-promoting effects of FGF19 on HCC cells.

## Materials and Methods

### Cell Culture and Treatments

The human HCC cell lines HepG2, Hep3B and PLC/PRF5 were obtained from the ATCC and grown as reported [20], [34]. The human HCC cell line Huh-7 (0403) was obtained from the Japanese Collection of Research Bioresources (JCRB, Tokyo, Japan) and maintained according to their instructions. Hep3B, PLC/PRF5, Huh-7 and SK-Hep1 cells do not contain mutations in the β-catenin gene, while HepG2 cells have a heterozygous deletion of 348 nucleotides in exon 3 of the β-catenin gene which results in the expression of an active truncated and more stable protein along with the wild type form. Where indicated cells were treated with the glycogen synthase kinase 3β (GSK3β) inhibitors LiCl and SB-415286 (Sigma, St. Louis, MO); the EGFR inhibitor PD153035 (Calbiochem, San Diego, CA); the AR goat polyclonal neutralizing antibody (αAR) (#AF262) or the control goat IgG (R&D Systems, Minneapolis, MN).

### Plasmid Constructs and Transfections

A 1255 bp fragment of human *AR* gene 5′ region, immediately upstream of the 5′ end of the mRNA start site, nucleotides −210 to −1464 from the ATG [35], was amplified from HepG2 cells genomic DNA using the primers described in Table S1. This *AR* 5′ DNA fragment was cloned upstream from the luciferase reporter gene in the pGL3-Basic vector (Promega, Madison, WI). Site-directed mutagenesis of the Tcf-binding elements (TBEs) was performed on this construct using the Quick Change Site-Directed Mutagenesis Kit (Stratagene, La Jolla, CA) according to the protocol supplied by the manufacturer. Mutations were as follows: TBE1: CTTTGTA → CTTTGCC; TBE2: CTTTGAA → CTTTGGC; TBE3: TACAAAC → GCCAAAG. The primers used for site-directed mutagenesis are described in Table S1. The expression vector for the dominant stable β-catenin T41A mutant and the dominant negative ΔNTcf4 have been described before and were the generous gift of Dr. Marie-Annick Buendia (Pasteur Institute, Paris, France) [36]. Cells were transiently transfected with the wild type and mutant AR-promoter reporter constructs, the empty pGL3-Basic vector as control, the TOPflash Tcf reporter plasmid (Millipore, Billerica, MA) as previously described [20]. Cells were also co-transfected with the Renilla luciferase reporter vector (Promega) as an internal control for transfection efficiency. Luciferase activity in cell lysates was determined using the Dual-Luciferase Reporter Assay System from Promega. Huh7 cells were stably transfected with the expression vector for β-catenin T41A mutant, and the empty expression vector (pcDNA3), and selected with G418 (Sigma) as described [20].

### RNA Isolation, Quantitative Real-time PCR (qPCR)

Total RNA was extracted using the TRI Reagent (Sigma). qPCR was performed using an iCycler (BioRad, Hercules, CA) and the iQ SYBR Green Supermix (BioRad) as reported [35]. Gene expression was determined using the ΔΔCT calculation as described [37], [38]. We designed all primers to distinguish between genomic and cDNA amplification and sequenced all PCR products to confirm the specificity. The sequences of primers used for qPCR are provided in Table S2.

### Chromatin Immunoprecipitation (ChIP)

The ChIP assay was performed in Huh7 cells essentially as described [22], using anti-β-catenin (#v9581) (Cell Signalling, Danvers, MA) or anti-Tcf4 (#05-511) (Millipore) antibodies, or a control rabbit IgG (Santa Cruz Biotechnology, Santa Cruz, CA). After ChIP, DNA was purified and the *AR* 5′ regions −1459 to −1287, −987 to −811 and −600 to −364, which include three putative Tcf/Lef binding sites previously identified by DNA sequence analysis [39], were PCR anplified and analyzed by agarose gel electrophoresis, or were analyzed by qPCR (qChIP) with the primers described in Table S3. Immunoprecipitations with IgG control antibody gave no amplification products upon PCR. Values were normalized to average values of inputs.

### Western Blot Analysis

Cells were homogenized and analyzed by Western blotting as described [22]. The antibodies used were: anti-phospho-GSK3β (Ser9) (#9336) from Cell Signaling, anti-active β-catenin (β-catenin dephosphorylated on Ser37 or Thr41) (#05-665) and anti-Tcf4 (#05-511) (Millipore). Polyclonal anti-β-actin antibody (#A2066) was from Sigma.

### RNA Interference

The sequences of the siRNAs targeting different regions of the human AR gene (siAR1 and siAR2), and the control siRNA (siGL2), along with their specificity have been described [20], [40]. siRNAs were from Sigma. Transfections of the siRNA duplexes (100 nM) were performed with the Lipofectamine RNAiMAX reagent (Invitrogen) following manufacturer’s instructions.

### AR Protein Determination by ELISA

Soluble AR was measured in cells’ conditioned medium by ELISA. Cells were cultured in the absence of serum as indicated, conditioned medium was collected and after addition of phenylmethylsulfonyl fluoride (1 mM) it was pre-cleared by centrifugation and lyophilized. The concentration of AR was determined by a sandwich ELISA from R&D Systems, using a monoclonal anti-AR capture antibody (#MAB262) and a biotinylated polyclonal detection antibody (BAF262). A standard curve using recombinant human AR (Sigma) was used to calculate AR concentrations in the conditioned medium.

### Immunofluorescence Staining

Cells were cultured on coverslips. After treatments cells were fixed with 4% paraformaldehyde (15 min) and subsequently permeabilized with 0.1% Triton X-100. After blocking in Supper blocking Buffer (Biorad, Hercules, CA) (30 min) fixed cells were incubated with anti-β-catenin antibodies (Santa Cruz Biotechnology, #sc-7963) (1∶100 in 1% albumin) overnight at 4°C. Slides were washed with saline and incubated with Alexa Fluor 488-conjugated rabbit anti-mouse secondary antibody (1∶500 in 1% abumin) (Invitrogen, Carlsbad, CA) for 1½ h at room temperature, and then washed and mounted.

### Data Mining in Gene Expression Datasets from Human HCC Tissues

Data mining was performed using public high-throughput gene expression datasets from human HCC tissues obtained from Gene Expression Omnibus (GEO) database. AR and FGF19 expression values were obtained from the following sets of data: GSE1898 (90 HCC tissue samples) [41] and GSE5975 (238 HCC tissue samples) [42].

### Statistical Analysis

Statistical analysis was performed with the Graph Pad Prism version 5.00 software (Graph Pad Software, San Diego, CA). Experiments were performed at least twice in triplicates. Data are means ± SEM. Data were compared among groups using the Student *t* test. The Spearman’s correlation coefficient was used to analyse the correlation between *AR* and *FGF19* gene expression values obtained from the above-mentioned datasets. A *P* value of <0.05 was considered significant.

## Results

### Activation of β-catenin Signaling Induces AR Gene Expression in Human HCC cells

**Figure 1:**
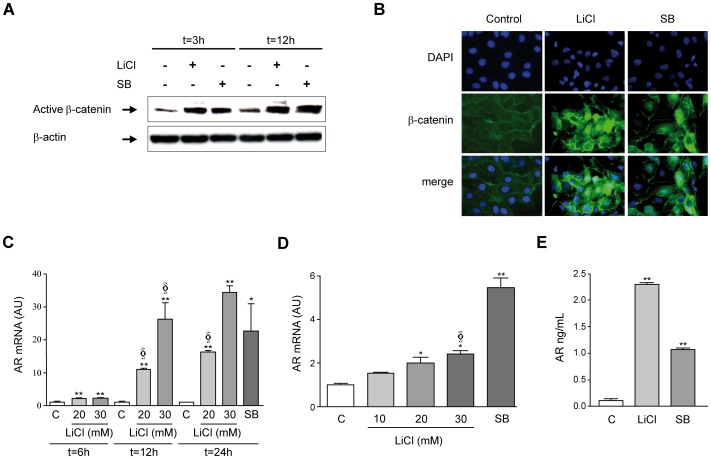
Activation of β-catenin signaling and AR gene expression in human HCC cells. (A) Huh7 cells were treated with LiCl (20 mM) or the GSK3β inhibitor SB-415286 (10 µM) (SB) for the indicated time points and active β-catenin was detected by immunoblotting with anti-dephosphorylated β-catenin specific antibodies. (B) Immunofluorescent demonstration of increased β-catenin protein levels and nuclear distribution in Huh7 cells treated with LiCl (20 mM) or SB-415286 (10 µM) (SB) for 12 h. Nuclei were stained with DAPI. Representative images are shown. (C) Time- and dose-dependent induction of *AR* gene expression in Huh7 cells upon β-catenin pathway activation by LiCl treatment. The effect of SB-415286 (10 µM) (SB) at 24 h of treatment is also shown. *P<0.05 *vs* control, **P<0.01 *vs* control, §*P*<0.05 *vs* same concentration of LiCl at the previous time. (D) Effect of β-catenin pathway activation by LiCl or SB-415286 (10 µM) (SB) treatment (24 h) on *AR* gene expression in Hep3B human HCC cells. *P<0.05 *vs* control, **P<0.01 *vs* control, §*P*<0.05 *vs* LiCl 10 mM. AU: arbitrary units. (E) Activation of β-catenin pathway induces AR protein secretion in human HCC cells. Huh7 cells were treated with LiCl (20 mM) or SB-415286 (10 µM) (SB) for 24 h in serum-free medium, and AR protein contents were determined by ELISA in the conditioned media. **P<0.01 *vs* control.

To examine the regulation of *AR* gene expression through the activation of the β-catenin signaling pathway we first used two well-known inhibitors of glycogen synthase kinase 3β (GSK3β), LiCl and SB-415286 [43], [44]. GSK3β is a component of the β-catenin destruction complex which phosphorylates β-catenin leading to its ubiquitination and degradation by the proteasomal pathway [26], [45]. Therefore GSK3β inhibition leads to β-catenin stabilization and accumulation. Accordingly, we found that upon LiCl or SB-415286 treatment levels of active (dephosphorylated) β-catenin increased in Huh7 cells (Figure 1A), correlating with the accumulation and nuclear translocation of β-catenin as evidenced by immunocytochemical staining (Figure 1B). Under these conditions we found that LiCl treatment resulted in a time- and dose-dependent increase in AR mRNA levels, which were also induced upon SB-415286 stimulation (Figure 1C). This effect was reproduced in Hep3B cells (Figure 1D), HepG2 cells (Fig. S1) and PLC/PRF5 cells (not shown). Consistent with the up-regulation of AR mRNA upon LiCl or SB-415286 treatment we detected increased levels of AR protein in the conditioned medium of Huh7 cells (Figure 1E).

**Figure 2:**
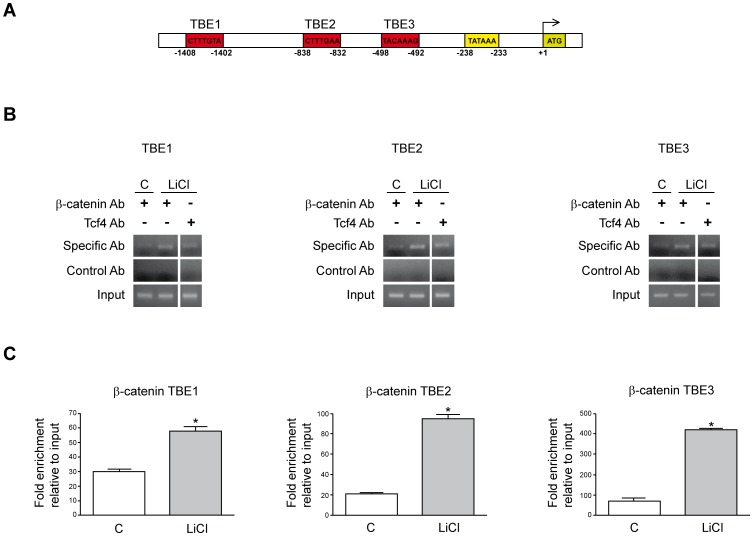
Activation of β-catenin pathway promotes β-catenin and Tcf4 recruitment to AR promoter in human HCC cells. (A) Identification in the human *AR* promoter sequence of three putative Tcf binding sites (TBE): TBE1, TBE2 and TBE3. The positions of these elements, and that of the TATA box, are indicated with numbering in reverse starting from the ATG. (B) ChIP assay evaluating the binding of β-catenin and Tcf4 to the three AR promoter regions encompassing the TBE1, TBE2 and TBE3 sites in control (C) and LiCl (20 mM, 24 h) treated Huh7 cells as indicated. Immunoprecipitated genomic DNA was PCR amplified and resolved in agarose gels. (C) DNA obtained after immunoprecipitation of chromatin with anti-β-catenin antibodies from control and LiCl treated cells was also quantified by qPCR as indicated in the Methods section. *P<0.05 *vs* control (C).

**Figure 3:**
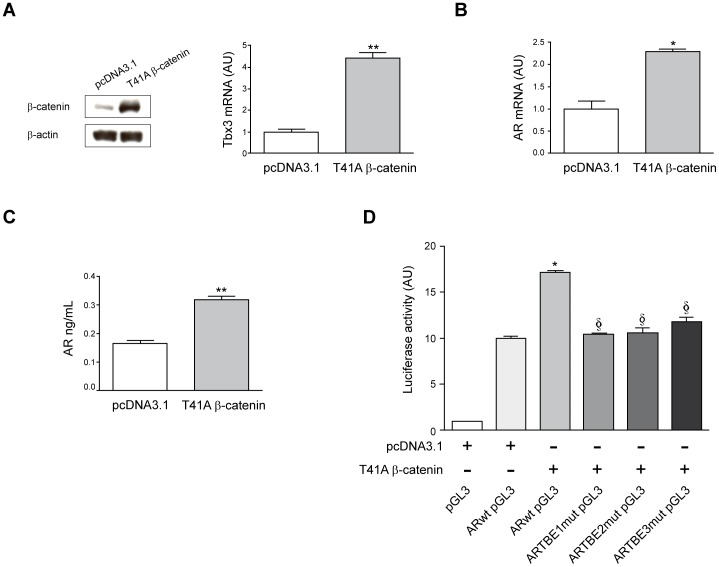
Expression of an active β-catenin mutant promotes AR gene expression in human HCC cells. (A) Left panel shows a representative Western blot of β-catenin protein levels in Huh7 cells stably transfected with an empty control vector (pcDNA3.1), or with the same vector harbouring a human stable β-catenin mutant (T41A β-catenin). Right panel shows the mRNA expression levels of the β-catenin target gene *Tbx3* in Huh7 cells stably transfected with control vector (pcDNA3.1) or with the T41A β-catenin active mutant. **P<0.01 *vs* control. (B) Expression levels of *AR* mRNA in Huh7 cells stably transfected with control vector (pcDNA3.1) or with the T41A β-catenin active mutant. *P<0.05 *vs* control. (C) AR protein contents as determined by ELISA in the conditioned media of Huh7 cells stably transfected with the control vector pcDNA3.1 or with the T41A β-catenin active mutant. Media was collected after 24 h culture in serum-free conditions. **P<0.01 *vs* control. (D) AR promoter activity is triggered by expression of the T41A β-catenin active mutant. Huh7 cells stably tansfected with the control vector pcDNA3.1 or with the T41 β-catenin active mutant, were transiently transfected with the empty luciferase reporter plasmid (pGL3) or with the wild type AR promoter luciferase reporter construct (ARwt pGL3). Huh7 cells expressing the T41A β-catenin active mutant were also transfected with three AR promoter constructs in which the three TBE sites had been individually mutated (ARTBE1mut pGL3, ARTBE2mut pGL3 and ARTBE3mut pGL3). Luciferase activity was measured as described in Methods. *P<0.05 *vs* ARwt pGL3-derived luciferase activity in pcDNA3.1 transfected cells. § *P*<0.05 *vs* ARwt pGL3-derived luciferase activity in T41A β-catenin transfected cells. AU: arbitrary units.

Activation of β-catenin signaling involves its nuclear translocation and interaction with members of the T-cell factor/lymphoid enhancer factor (Tcf/Lef) family of transcription factors, leading to the formation of regulatory complexes that bind Tcf-binding elements (TBEs) on the promoters of target genes [26], [46], [47]. A previous *in silico* study on the potential regulation of the expression of EGF family members by the Wnt/β-catenin pathway had identified three putative consensus TBE sites in the 5′ region of the human *AR* gene [39] (Figure 2A). To characterize the mechanisms involved in AR gene regulation by β-catenin signaling we examined the *in vivo* binding of β-catenin and Tcf4 to these TBEs (TBE1, TBE2 and TBE3). As shown in Figure 3B, ChIP analysis indicated that binding of β-catenin to these three TBEs was significantly increased upon LiCl treatment of Huh7 cells. Chromatin immunoprecipitation with a Tcf4 specific antibody also detected Tcf4 binding to the three TBEs (Figure 2B). The induction of β-catenin binding upon LiCl stimulation to TBEs in *AR* 5′ region was further confirmed by quantitative analysis of immunoprecipitated chromatin (qChIP) (Figure 2C). These findings indicated that Tcf4/β-catenin specifically bound to *AR* 5′ region under conditions of active β-catenin pathway signaling.

To further examine the involvement of β-catenin in the regulation of AR gene expression we stably transfected Huh7 cells with an active β-catenin mutant (β-catenin T41A) or the corresponding empty vector. This β-catenin T41A mutant protein has increased stability, and has been previously shown to significantly up-regulate the expression of β-catenin target genes [36], [48]. Consistently we found increased β-catenin protein levels in β-catenin T41A expressing Huh7 cells compared with control cells stably transfected with the empty expression vector, and increased expression of the Wnt/β-catenin target gene *Tbx3* [36] (Figure 3A). T41A β-catenin expressing Huh7 cells also displayed increased levels of AR mRNA and secreted more AR protein to the culture medium (Figure 3B and C). These observations confirmed the notion that β-catenin pathway activation can trigger AR gene expression in HCC cells. To gain further insight on the mechanisms involved in the regulation of AR gene transcription by β-catenin/Tcf, site-directed mutation of the TBE sites found in AR 5′ regulatory region was performed in the context of a −1255 bp *AR* promoter luciferase reporter plasmid, as described in Materials and Methods. Transfection of the wild type *AR* promoter construct in Huh7 cells expressing the active T41A β-catenin mutant resulted in a significant stimulation of AR promoter activity compared to cells transfected with the control vector pcDNA3.1 (Figure 3D). However, mutation in either TBE site markedly reduced T41A β-catenin-induced AR promoter activity (Figure 3D). Altogether, these evidences indicate that β-catenin pathway activation triggers AR gene expression in HCC cells and that *AR* would be a direct transcriptional target of the β-catenin/Tcf complex.

### β-Catenin Promotes HCC cell Proliferation through the Induction of AR

**Figure 4:**
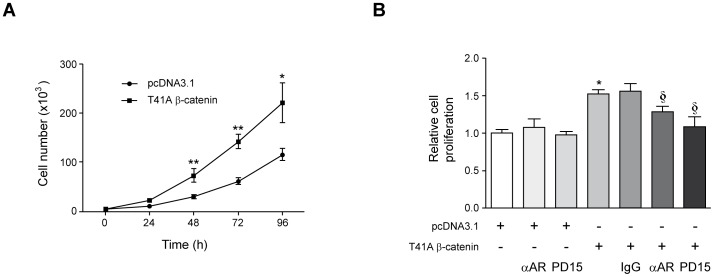
Activation of the β-catenin pathway promotes HCC cell growth involving AR/EGFR signaling. (A) Proliferation of Huh7 cells stably transfected with control vector pcDNA3.1 or the T41A β-catenin active mutant. Cells were grown in the presence of 10% fetal calf serum. *P<0.05 and **P<0.01 *vs* pcDNA3.1 transfected cells. (B) Proliferation induced by T41A β-catenin active mutant is attenuated by AR neutralizing antibodies or the EGFR inhibitor PD153035. T41A β-catenin expressing Huh7 cells were incubated in the absence or presence of AR neutralizing antibodies (αAR, 20 µg/mL), control purified goat IgG (IgG, 20 µg/mL), PD153035 (1 µM, PD15), for 72 h, and cell proliferation relative to pcDNA3.1 transfected cells was measured. The effect of these treatments on control pcDNA3.1 transfected cells is also shown. These treatments were performed in cells maintained in 10% fetal calf serum. *P<0.05 *vs* pcDNA3.1 transfected cells, § *P*<0.05 *vs* untreated T41A β-catenin transfected cells.

Signaling through the β-catenin pathway has been thoroughly demonstrated to contribute to HCC cell growth [36], [49], [50]. This is supported in part by the growth-promoting effects of constitutively active β-catenin mutants, such as S33Y, in human fetal hepatoblasts and HCC cells [51]. Here we also observed that Huh7 cells stably expressing the T41A β-catenin active mutant showed enhanced proliferation compared with cells transfected with the control empty vector (Figure 4A). Interestingly, the growth of T41A β-catenin expressing Huh7 cells was significantly reduced when cells were treated with the EGFR inhibitor PD153035 (83.7% reduction over the stimulation conferred by T41A β-catenin expression), or incubated in the presence of specific AR neutralizing antibodies (46.1% reduction) (Figure 4B). Combination of αAR and PD153035 did not further increase the already potent effect of the EGFR inhibitor (data not shown). The involvement of AR on the enhanced proliferation of T41A β-catenin expressing cells was additionally demonstrated upon AR gene expression knockdown with AR specific siRNAs (Fig. S2). These findings indicate that at least part of the growth-promoting effects of β-catenin pathway activation may be mediated in part through the up-regulation of *AR* gene expression.

### FGF19 Activates β-catenin Signaling and AR Gene Expression in Human HCC cells

**Figure 5:**
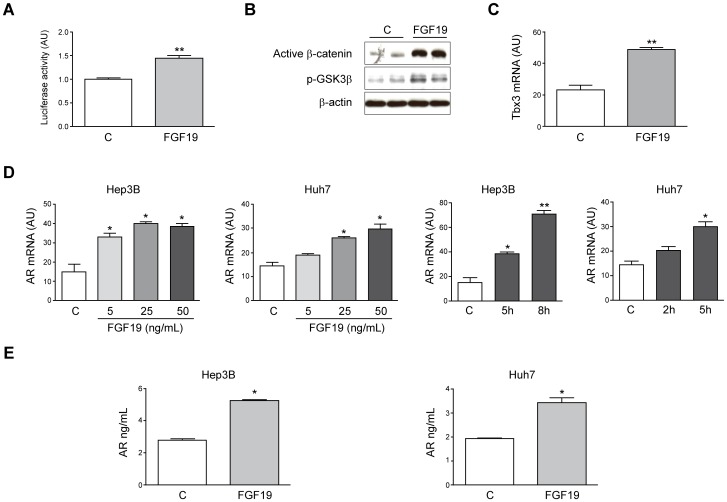
FGF19 activates β-catenin signaling and induces AR gene expression in human HCC cells. (A) Hep3B cells were transfected with a Tcf-regulated reporter vector and after 24 h were treated or not with FGF19 (50 ng/mL) for 5 h, then luciferase activity was measured as described in Methods. *P<0.05 *vs* control (C) cells. (B) Hep3B cells were treated or not with FGF19 (50 ng/mL, 10 min) and active β-catenin or phosphorylated GSK3β (p-GSK3β) were detected by Western blotting. Representative blots of two experiments performed in duplicates are shown. (C) Expression (mRNA levels) of the β-catenin target gene *Tbx3* in Hep3B cells treated or not with FGF19 (50 ng/mL, 5 h). **P<0.01 *vs* control. (D) Left panels: dose-dependent induction of AR expression (mRNA) in Hep3B and Huh7 cells treated with the indicated concentrations of FGF19 for 5 h. Right panels: time-dependent induction of AR expression (mRNA) in Hep3B and Huh7 cells treated with 50 ng/mL of FGF19. *P<0.05 *vs* control, **P<0.01 *vs* control (C). (E) AR protein contents were determined by ELISA in the conditioned media of FGF19 treated (50 ng/mL, 24 h) or control (C) Hep3B and Huh7 cells. *P<0.05 *vs* control cells. AU: arbitrary units.

Aberrant activation of the Wnt/β-catenin pathway in HCC can be accounted for by mutations in the *CTNNB1* gene as well as by altered expression of ligands, receptors and inhibitors of the Wnt pathway [26], [27]. In addition, β-catenin signaling can be activated through cross-talks with other signaling pathways deregulated in HCC [26], [33], [52]–[54]. Among these pathways the one driven by FGF19, and its tyrosine kinase receptor FGFR4 and co-receptor β-klotho, is perhaps the best characterized in its functional interaction with the β-catenin system in HCC cells [32]. Activation of β-catenin by FGF19 has been already shown in Huh7 cells [32], here we extend this response to Hep3B cells. Interestingly, compared to other HCC cell lines Hep3B cells expresses higher basal levels of FGF19, FGFR4, β-klotho and AR mRNA (Fig. S3). As shown in Figure 5A, treatment of Hep3B cells with FGF19 increased luciferase activity in cells transfected with Tcf-luciferase reporter plasmid. Upon FGF19 treatment, cells also displayed increased levels of active (dephosphorylated) β-catenin and phospho-GSK3β as analyzed by immunoblotting (Figure 5B), and elevated mRNA levels of the Wnt/β-catenin target gene *Tbx3* [36](Figure 5C). Next we evaluated if FGF19 could affect *AR* gene expression in HCC cells. As shown in Figure 5D, FGF19 treatment resulted in a time- and dose-dependent increase in AR mRNA levels, and enhanced accumulation of AR protein in the cells’ conditioned media (Figure 5E).

**Figure 6:**
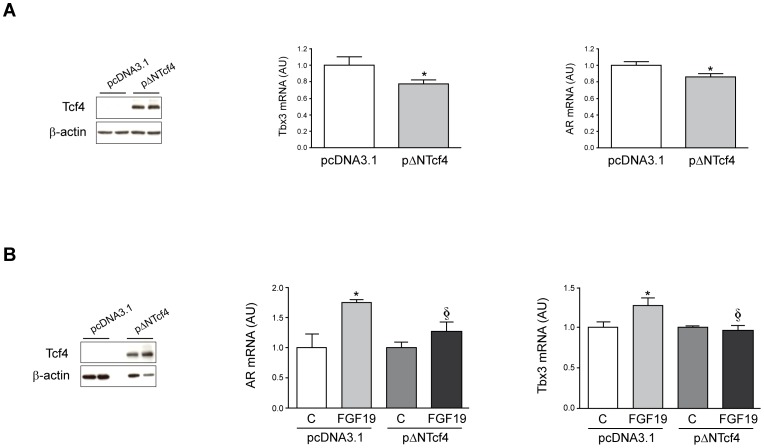
Inhibition of β-catenin activity by dominant negative Tcf4 (ΔNTcf4) reduces basal and FGF19-induced AR gene expression in HCC cells. (A) HepG2 cells, that express an active mutant β-catenin allele, were transiently transfected with the control pcDNA3.1 vector or with this vector harbouring ΔNTcf4 cDNA. Left panel demonstrates Tcf4 protein levels in control and ΔNTcf4 transfected HepG2 cells analyzed by Western blotting 24 h after transfections. In these cells the mRNA levels of the β-catenin target gene *Tbx3* (central panel) and those of *AR* (right panel) were also analyzed. *P<0.05 *vs* cells transfected with pcDNA3.1. (B) Huh7 cells were transiently transfected with the control pcDNA3.1 vector or with this vector harbouring ΔNTcf4 cDNA. Left panel shows Tcf4 protein levels in control and ΔNTcf4 transfected Huh7 cells analyzed by Western blotting 24 h after transfections. Transfected cells were then treated or not with FGF19 (50 ng/mL, 5 h), and the mRNA levels of the β-catenin target gene *Tbx3* (central panel) and those of *AR* (right panel) were measured. *P<0.05 *vs* control cells (C) transfected with pcDNA3.1. §*P*<0.05 *vs* cells transfected with pcDNA3.1 and treated with FGF19.

To evaluate the role played by the β-catenin system in FGF19-mediated AR expression, we made use of a previously reported dominant negative ΔNTcf4 expression vector [36]. First we transfected this expression vector in HepG2 cells, which express a more stable truncated β-catenin allele [50], [55]. As shown in Figure 6A, expression of ΔNTcf4 resulted in reduced basal expression of the Wnt/β-catenin target gene *Tbx3* and also in decreased AR mRNA levels, confirming the biological activity of this dominant negative Tcf4 mutant and the regulation of *AR* gene expression in response to the manipulation of the β-catenin pathway. Next we examined the response of ΔNTcf4 expressing HCC cells to FGF19 treatment. We observed that the up-regulation of AR expression in Huh7 cells treated with FGF19 was attenuated in cells expressing ΔNTcf4 compared to cells transfected with control plasmid, and a similar response was observed regarding *Tbx3* expression (Figure 6B).

**Figure 7:**
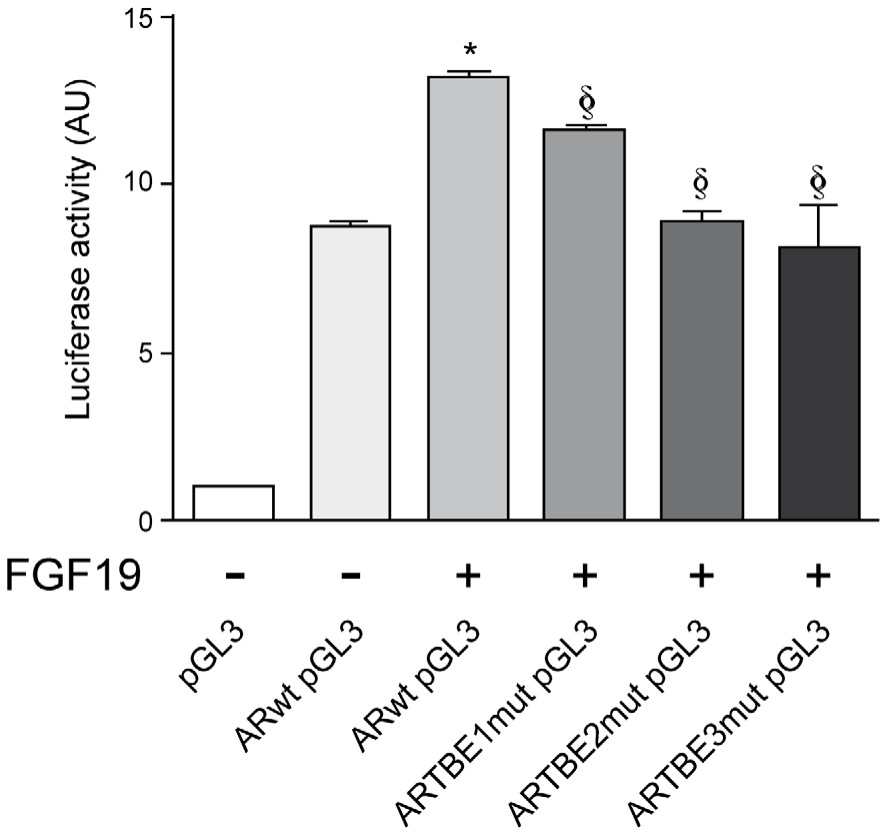
Transactivation of AR promoter by FGF19 treatment depends on the integrity of TBE sites. Hep3B cells were transiently transfected with the empty luciferase reporter plasmid (pGL3), with the wild type AR promoter luciferase reporter construct (ARwt pGL3) and with the three AR promoter constructs in which the three TBE sites had been individually mutated (ARTBE1mut pGL3, ARTBE2mut pGL3 and ARTBE3mut pGL3). Cells were treated as indicated with FGF19 (50 ng/mL, 12 h) and luciferase activity was measured as described in Methods. *P<0.05 *vs* untreated ARwt pGL3 transfected cells. §*P*<0.05 *vs* ARwt pGL3 transfected cells treated with FGF19. AU: arbitrary units.

To further evaluate the implication of β-catenin signaling on the FGF19-mediated regulation of *AR* gene expression, we examined the response of the above-described wild type *AR* promoter luciferase reporter construct and the three TBE mutants to FGF19 treatment. As shown in Figure 7, FGF19 stimulation resulted in a significant increase in AR promoter activity. In line with our previous findings in T41A β-catenin active mutant expressing cells, the FGF19-triggered activation of *AR* promoter was markedly reduced when either TBE site was mutated.

### Functional Relevance of AR Up-regulation in FGF19-induced HCC cell Proliferation

**Figure 8:**
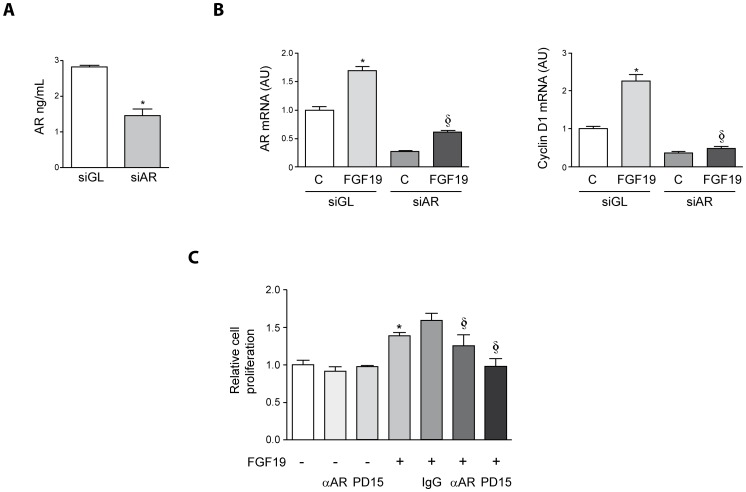
AR expression is required for FGF19 mediated cyclin D1 induction and HCC cell proliferation. (A) Hep3B cells were transfected with AR specific siRNAs (siAR) or control siRNAs (siGL). AR protein concentration in conditioned medium from siGL and siAR transfected Hep3B cells was determined by ELISA 72 h after transfections. (B) Hep3B cells were transfected with control siRNAs (siGL) or AR specific siRNAs (siAR) and 24 h later cells were treated with FGF19 (50 ng/mL, 5 h) as indicated.*P<0.05 *vs* siGL transfected control (C) cells. AR (left panel) and cyclin D1 (right panel) gene expression were measured by qPCR. §*P*<0.05 *vs* siGL transfected and FGF19 treated cells. AU: arbitrary units. (C) Proliferation induced by FGF19 is attenuated by AR neutralizing antibodies or the EGFR inhibitor PD153035. Hep3B cells were incubated with FGF19 (50 ng/mL) as indicated in the absence or presence of AR neutralizing antibodies (αAR, 20 µg/mL), control purified goat IgG (IgG, 20 µg/mL) or PD153035 (1 µM, PD15) for 48 h, and cell proliferation relative to untreated cells was measured. The effect of αAR and PD153035 on control (FGF19 untreated) cells is also shown. All treatments were performed on cells kept in medium containing 5% fetal calf serum. *P<0.05 *vs* untreated cells. §*P*<0.05 *vs* FGF19 treated cells.

FGF19 has been recently identified as a new growth factor capable of inducing the proliferation of HCC cells and as a driver gene in hepatocarcinogenesis [32], [56], [57]. Induction of cyclin D1 expression by FGF19 involves the activation of the β-catenin pathway, and was found to be essential for the promotion of HCC cell growth [32]. Considering that AR is a mitogenic factor for HCC cells [20], [21], and an FGF19 target gene according to our current findings, we now examined the role of FGF19-induced AR in HCC cell proliferation. To do so we knocked down AR expression by transfecting Hep3B cells with AR-specific or control siRNAs prior to FGF19 stimulation. Figure 8A shows the effect of AR-specific siRNA transfection on AR protein concentration in Hep3B cells conditioned medium. As shown in Figure 8B, the AR mRNA levels elicited by FGF19 treatment were effectively reduced upon transfection with AR-specific siRNAs when compared with those induced in cell transfected with control siRNAs (siGL). Interestingly the stimulation of cyclin D1 gene expression induced by FGF19 (two-fold over controls) was blunted when AR mRNA levels were knocked down. This suggested that AR would be an important mediator of FGF19 effects on the expression of this key cell cycle regulatory gene. To further explore the role of AR in the proliferative effects of FGF19 we measured the growth of Hep3B cells treated with FGF19 in the presence of specific AR neutralizing antibodies. Under these conditions we could demonstrate a significant reduction in FGF19-induced cell proliferation (88.7% inhibition of the growth induced by FGF19 treatment) that was not observed in cells incubated with control IgGs (Figure 8C). A role for AR/EGFR signaling in FGF19-mediated cell proliferation was further supported by the inhibitory effects of the EGFR inhibitor PD153035, which reduced cell growth even below the levels found in untreated cells (Figure 8C). The inhibitory effects of AR neutralizing antibodies and PD153035 on basal Hep3B cell proliferation previously reported [20] were not clearly observed in this experiment (Figure 8C). This is likely due to the fact that the cell proliferation experiments shown here were performed in cells kept in serum-containing medium (5% serum), as described by Miura et al. [57]. However, previous studies were carried out in serum-free conditions, under which dependence on autocrine AR for HCC proliferation is magnified [20].

### Correlation between FGF19 and AR Gene Expression in Human HCC Tissues

**Figure 9:**
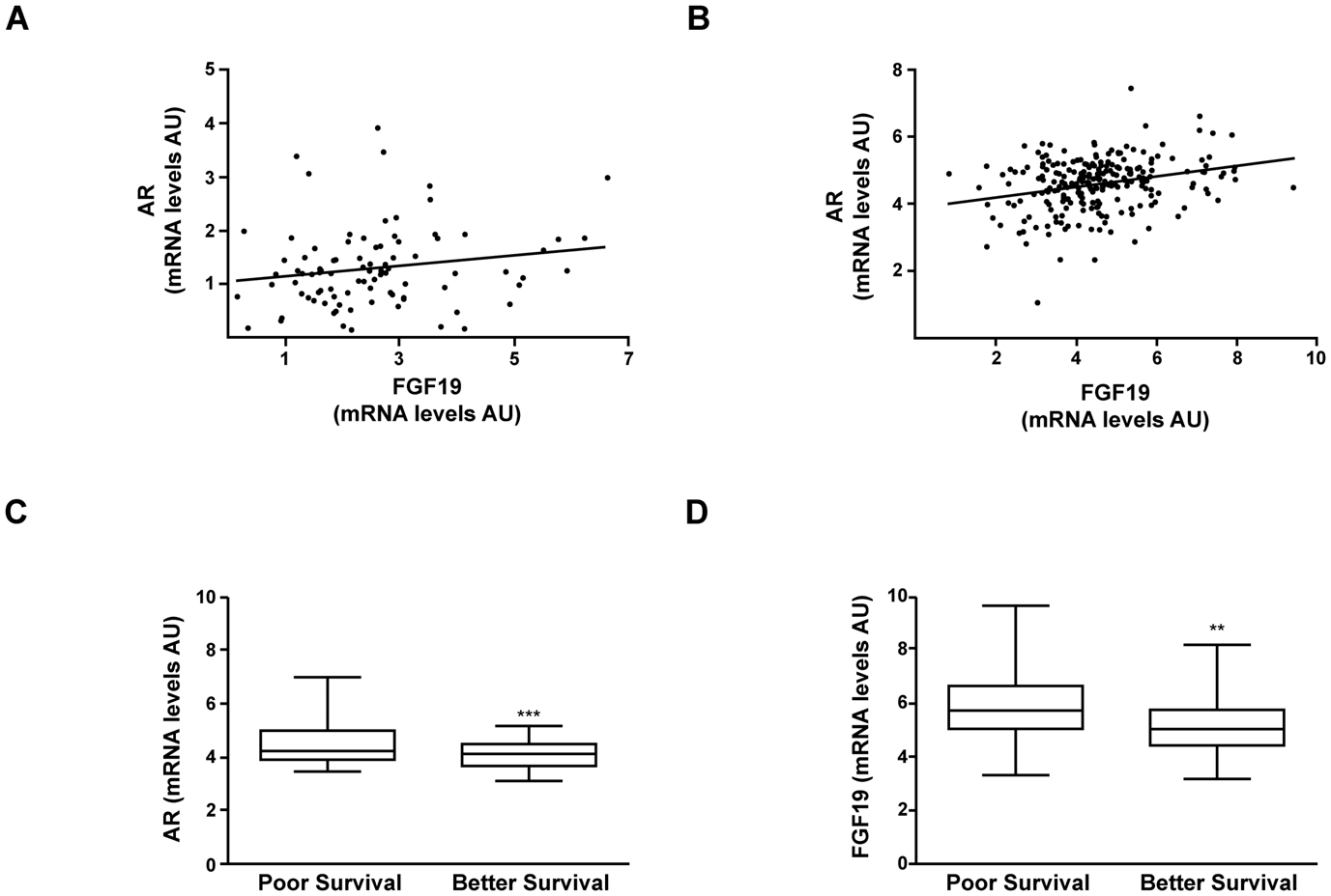
AR and FGF19 gene expression show a positive correlation in human HCC tissue samples and are associated with poor patient prognosis. Gene expression data was obtained for *AR* and *FGF19* from two independent microarray studies and correlation of expression levels was analyzed as described in Methods. (A) *AR* and *FGF19* gene expression values were obtained from the GSE1898 dataset (90 HCC tissue samples) and showed a significant positive correlation r = 0.18, *P*<0.05. (B) AR and FGF19 gene expression values were obtained from the GSE5975 dataset (238 HCC tissue samples) and showed a positive correlation r = 0.25, *P*<0.0001. (C) *AR* and *FGF19* gene expression in the subclasses of tumors corresponding to patients with poor and better survival originally identified in the GSE1898 dataset. ***P = 0.0005, **P = 0.0068.

To further support our present experimental observations on the regulation of AR gene expression by FGF19 in human HCC cell lines we analyzed previously published microarray data from two independent gene expression profiling studies. Data was available from Gene Expression Omnibus (GEO) database for 90 and 239 HCC tumor tissues respectively [41], [42]. As shown in Figure 9A and B, our data-mining approach revealed a significant positive correlation between FGF19 and AR mRNA levels in these ample groups of human HCC tissues. In addition, we also examined the expression of AR and FGF19 in the two subclasses of tumours with differential prognosis that were identified in the GSE1989 data set according to their gene expression profiles [41]. Interestingly, we found that the expression of AR and FGF19 genes was higher in the subclass of tumours with strong cell proliferation and anti-apoptosis gene expression signatures, corresponding to patients with poorer prognosis (reduced survival) [41] (Figure 9 C).

## Discussion

The EGFR ligand AR is increasingly recognized as a potent oncogenic factor over-expressed in a variety of human cancers including HCC [23], [58]. AR has been demonstrated to participate in most of the characteristic traits of transformed cells and therefore constitutes a promising therapeutic target. Anti-EGFR monoclonal antibodies and direct inhibition of the EGFR tyrosine kinase activity are among the most explored anti-cancer targeted therapies [12]–[14]. However, these strategies have not always translated into efficacious clinical responses [7], [13], [14], [59]. Down-regulation of AR expression and availability, as well as the use of AR-neutralizing antibodies, have shown promising pre-clinical results, suggesting that these strategies could enhance the effectiveness of other targeted and conventional antitumoral approaches [23], [25], [58], [60]. To this end, understanding the mechanisms leading to AR overexpression in cancer cells, including HCC cells, is an important albeit unresolved issue [23]. Through different complementary approaches we demonstrate in this study that *AR* gene expression can be activated in human HCC cells by β-catenin signaling, a key signaling pathway in hepatocarcinogenesis [11]–[15], [26], [27]. We provide evidence showing that AR is a transcriptional target of the β-catenin/Tcf4 complex, including the demonstration of direct binding of β-catenin and Tcf4 to three putative TBE sites in the *AR* promoter region upon β-catenin pathway activation. Endogenous *AR* expression and secretion to the extracellular medium was induced in HCC cells in response to the ectopic expression of the dominant stable β-catenin mutant T41A [36]. Conversely, AR mRNA levels, together with those of the well-characterized β-catenin target gene *Tbx3* [36], were reduced by the expression of a dominant negative Tcf4 variant (ΔNTcf4) in HepG2 cells, which express a stable β-catenin allele [36], [50], [51]. Furthermore, *AR* promoter-reporter activity triggered by the expression of this active β-catenin variant was reduced upon mutation of the TBE sites within this *AR* 5′ region. Together, these observations strongly suggest that AR is a target of β-catenin signaling in human HCC cells. Nevertheless, β-catenin signaling activates a wide variety of target genes that putatively could be involved in HCC tumorigenesis and malignant behaviour [26], [36], [47], [61]. In this context, establishing the functional significance of β-catenin-stimulated AR gene expression was therefore important. Expression of active β-catenin variants such as the S33Y mutant in Huh7 cells has been shown to increase their growth [47]. Here we demonstrate that the T41A β-catenin mutant also enhanced Huh7 cell proliferation. Interestingly, proliferation of T41A β-catenin expressing Huh7 cells was partially reduced when EGFR activity was inhibited with PD153035, and in the presence of specific AR-neutralizing antibodies. These findings demonstrate that increased AR expression in response to β-catenin pathway activation indeed plays a role in the growth elicited by enhanced β-catenin signaling in HCC cells.

Functional cross-talk between Wnt-triggered signaling and the EGFR system has been previously identified in other cancer cells of epithelial origin such as breast cancer cells [62]. In these cells Wnt signaling increased the availability of EGFR ligands, leading to EGFR transactivation, cyclin D1 expression and cell proliferation [28], [63]. However, these effects occurred through mechanisms independent from the canonical Wnt signaling pathway, and were linked to Src-mediated activation of the metalloproteases that mediate the release of EGFR ligands from the cell surface [63]. Moreover, a recent study demonstrated that the high endogenous expression of AR in breast cancer cells potently and constitutively down-regulates the expression of Dikkopf1, a secreted inhibitor of the Wnt/β-catenin pathway [64]. These findings further illustrate the multifarious interactions between the Wnt/β-catenin and EGFR systems in cancer cells. Our current observations provide a novel point of convergence between EGFR and β-catenin pathways in human HCC cells, in this case through the regulation of the expression of the EGFR ligand gene *AR* by β-catenin signaling.

As previously mentioned several mechanisms account for β-catenin pathway activation in HCC cells, and these include mutations in β-catenin gene (*CTNNB1*) or in components of the β-catenin degradation complex (*AXIN1*), as well as dysregulation in the expression of Wnt/Fizzled signaling elements [11], [26], [27], [47], [55], [61]. In addition, signaling pathways commonly activated in HCC cells such as those triggered by FGF19, TGFβ, or the HGF receptor MET, have been reported to stimulate β-catenin signaling [32], [53], [65]. Activation of β-catenin through the FGF19/FGFR4 system has been thoroughly characterized in human HCC and colon cancer cells [32], [33]. This, together with previous evidences showing the ability of the closely-related FGFR1 receptor to up-regulate *AR* expression in epithelial cells [66], led us to examine whether *AR* could be a target of FGF19/FGFR4 signaling. Here we first demonstrate that FGF19 is a novel inducer of *AR* gene expression in human HCC cells. Moreover, the inhibitory effect of dominant negative ΔNTcf4 on FGF19 induced AR expression, as well as the attenuated response to FGF19 of *AR* promoter-reporter constructs with mutated TBE sites, strongly suggest that β-catenin signaling is involved in this response. *FGF19* has been recently identified as a driver gene in human hepatocarcinogenesis [32]. FGF19 gene expression is elevated in liver tumors correlating with a poor prognosis, and FGF19 neutralizing antibodies inhibit experimental HCC growth [56], [57]. In HCC cells FGF19 signals through the FGFR4/β-klotho complex, which is also frequently up-regulated in human HCC tissues and is emerging as a good candidate anti-tumor target [66]–[69]. Although the intracellular pro-tumorigenic mechanisms triggered by FGF19/FGFR4/β-klotho signaling are not completely known induction of cyclin D1 expression is considered a key event, and this effect also involved the activation of β-catenin signaling [32]. Interestingly, we found that knockdown of AR expression blunted the induction of cyclin D1 mediated by FGF19 in HCC cells. In line with this finding we also observed that upon EGFR inhibition, or in the presence of AR neutralizing antibodies, the proliferative effects of FGF19 on HCC cells were significantly attenuated. Taken together our results identify AR as a new downstream target of FGF19 in HCC cells, and reveal a functional cross-talk in which the AR/EGFR system could mediate, and likely amplifiy, the tumor-promoting effects of FGF19. As mentioned above FGF19 expression is increased in a subset of human HCCs with poor prognosis [57]. Our finding that *AR* and *FGF19* gene expression showed a positive correlation in human HCC tissues suggests that to a certain extent *AR* gene expression could be modulated by FGF19 in liver tumors, and supports the clinical relevance for our experimental observations. Moreover, the increased expression of these two genes in a subclass of tumors corresponding to patients with poorer prognosis [41] suggests their potential contribution to the progression of the disease. From a translational point of view, our observations also suggest that the efficacy of FGF19/FGFR4 targeted therapies that are currently being devised for HCC treatment [56], [66], [69] could be enhanced by simultaneously interfering with the AR/EGFR system.

## Supporting Information

Figure S1 **AR gene expression in HepG2 cells upon β-catenin signalling activation.** HepG2 cells were treated with LiCl (12 h) or the GSK3β inhibitor SB-415286 (SB, 10 µM) (24 h) and AR mRNA levels were determined. *P<0.05 *vs* control (C), §*P*<0.05 *vs* cells treated with LiCl 20 mM. AU: arbitrary units.
(TIF)

Figure S2 **Knockdown of AR gene expression reduces the proliferation of Huh7 cells expressing mutant β-catenin**. T41A β-catenin expressing cells and control pcDNA3.1 transfected cells were transfected with AR specific siRNA (siAR) or control siRNA (siGL). Cells were counted at the indicated time-points 24 h after transfections. *P<0.05 *vs* siAR transfected cells. At 72 h knockdown of AR expression in T41A β-catenin cells reduced cell proliferation by 45%, while in control pcDNA3.1 transfected cells proliferation was reduced by 26%.
(TIF)

Figure S3 **Comparative analysis of AR, FGF19, FGFR4 and β-klotho (KLB) mRNA levels in Huh-7, Hep3B and HepG2 cells.** Gene expression was analyzed by qPCR. AU: arbitrary units.
(TIF)

Table S1 **Primers used for amplification of human** ***AR*** **gene 5′ region genomic DNA.**
(DOC)

Table S2 **Primers used in this study for qPCR analysis of gene expression.**
(DOC)

Table S3 **Primers used for qPCR analysis of the** ***AR*** **gene promoter region encompassing the three studied TBE sites.**
(DOC)
