# Supplementary figures and images for: Regulation of Amphiregulin Gene Expression by β-Catenin Signaling in Human Hepatocellular Carcinoma Cells: A Novel Crosstalk between FGF19 and the EGFR System
Source: PLoS One. 2012 Dec 20;7(12):e52711. doi: 10.1371/journal.pone.0052711 (PMC3527604; doi:10.1371/journal.pone.0052711)

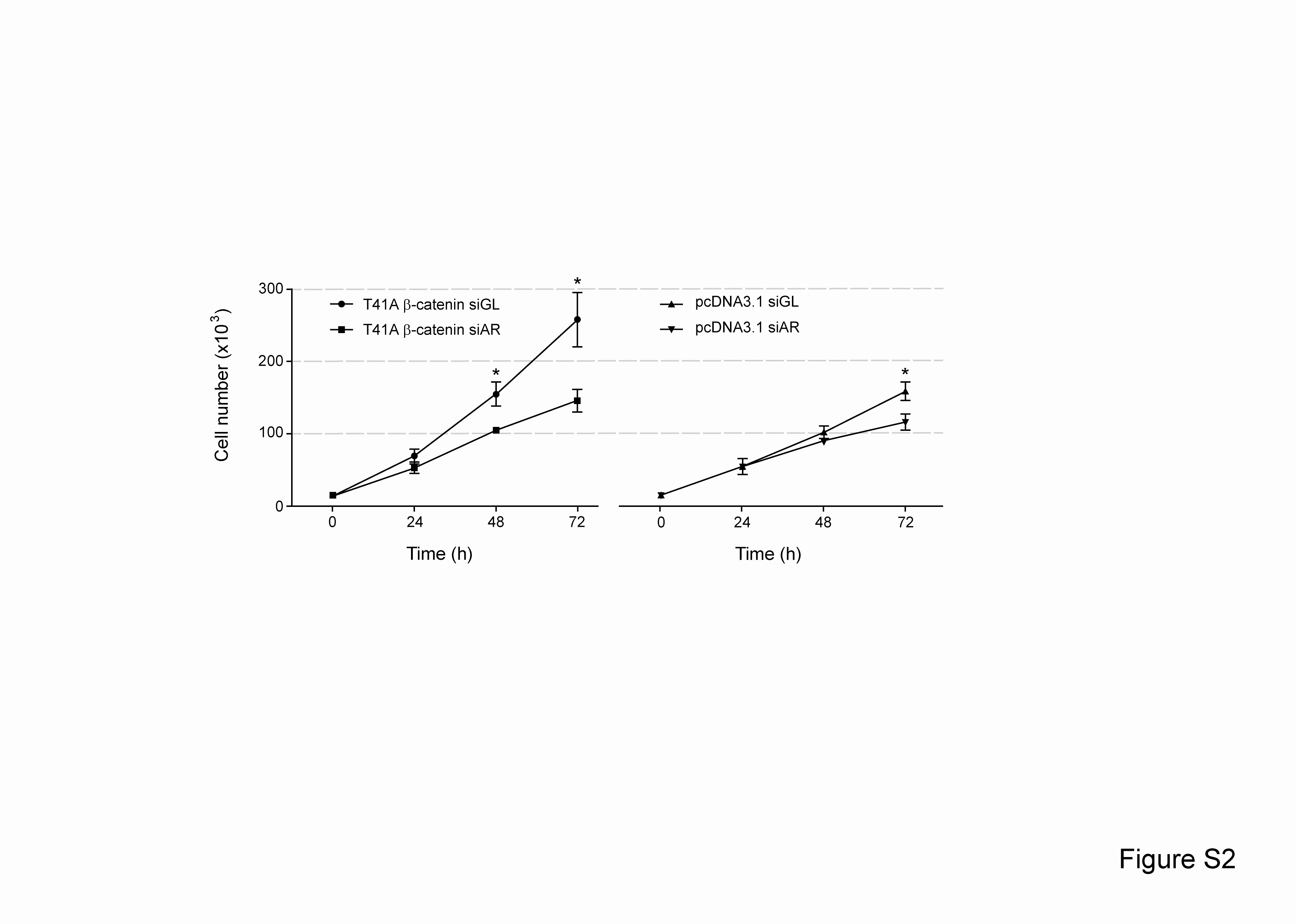

Supplement: Figure S2 — Knockdown of AR gene expression reduces the proliferation of Huh7 cells expressing mutant β-catenin. T41A β-catenin expressing cells and control pcDNA3.1 transfected cells were transfected with AR specific siRNA (siAR) or control siRNA (siGL). Cells were counted at the indicated time-points 24 h after transfections. *P<0.05 vs siAR transfected cells. At 72 h knockdown of AR expression in T41A β-catenin cells reduced cell proliferation by 45%, while in control pcDNA3.1 transfected cells proliferation was reduced by 26%. (TIF) [file pone.0052711.s002.tif]
