# Supplementary material for: Regulation of Amphiregulin Gene Expression by β-Catenin Signaling in Human Hepatocellular Carcinoma Cells: A Novel Crosstalk between FGF19 and the EGFR System
Source: PLoS One. 2012 Dec 20;7(12):e52711. doi: 10.1371/journal.pone.0052711 (PMC3527604; doi:10.1371/journal.pone.0052711)
Supplement: Table S1 — Primers used for amplification of human AR gene 5′ region genomic DNA. (DOC) [file pone.0052711.s004.doc]

**Supplementary Table S1.**

- Primers used for amplification of human *AR* gene 5’ region genomic DNA.

Nucleotides -1464 to -210 from the ATG.

Sense 5’-CAGCCCACCCGAGTAGCTGGGACTA-3’

Antisense 5’-GTAGGGCGGCGCGCACCTGCCGCTTTATA-3’

- Primers used for site-directed mutagenesis of TBE sites in the cloned *AR* gene 5’ region genomic DNA.

TBE1 site sense (5’-3’)

TCTGTTGTAGATGTTAAGTA**GC**CAAAGAGGTTGTCAGAGTTTGAAAC

TBE1 site antisense (5’-3’) GTTTCAAACTCTGACAACCTCTTTG**GC**TACTTAACATCTACAACAGA

TBE2 site sense (5’-3’)

AAGAATTCATATCCACCTGGCTTTG**GC**CATTATCGGCTGTGAGATGG

TBE2 site antisense (5’-3’)

CCATCTCACAGCCGATAATG**GC**CAAAGCCAGGTGGATATGAATTCTT

TBE3 site sense (5’-3’) CATCACGCCCAGCTAATTTC**C**TTTG**GC**TTTTTAGTAAAGATGGGGTT

TBE3 site antisense (5’-3’) AACCCCATCTTTACTAAAAA**GC**CAAA**G**GAAATTAGCTGGGCGTGATG
