# Supplementary material for: Regulation of Amphiregulin Gene Expression by β-Catenin Signaling in Human Hepatocellular Carcinoma Cells: A Novel Crosstalk between FGF19 and the EGFR System
Source: PLoS One. 2012 Dec 20;7(12):e52711. doi: 10.1371/journal.pone.0052711 (PMC3527604; doi:10.1371/journal.pone.0052711)
Supplement: Table S2 — Primers used in this study for qPCR analysis of gene expression. (DOC) [file pone.0052711.s005.doc]

**Supplementary Table S2.**

Primers used in this study for qPCR analysis of gene expression.

AR S CTGTCGCTCTTGATACTCGG

AS GCCAGGTATTTGTGGTTCGT

Tbx3 S GAAGAAGAGGTGGAGGACGA

AS GACATCCACTGTTCCCCAGT

Cyclin D1 S TGGAACACCAGCTCCTGTGC

AS TCCAGGTAGTTCATGGCCAG

FGFR4 S GCTGGCTTAAGGATGGACAG

AS CGTTGATGACGATGTGCTTC

β-Klotho S AAGACACCACGGCCATCTAC

AS ATTCAGTGACACCCCAGGAG

FGF19 S CTCTCCAGCTGCTTCCTGCGCATC

AS TTGTAGCCATCAGGGCGGATCTCC

Histone H3 S AAAGCCGCTCGCAAGAGTGCG

AS ACTTGCCTCCTGCAAAGCAC
