# Supplementary material for: Regulation of Amphiregulin Gene Expression by β-Catenin Signaling in Human Hepatocellular Carcinoma Cells: A Novel Crosstalk between FGF19 and the EGFR System
Source: PLoS One. 2012 Dec 20;7(12):e52711. doi: 10.1371/journal.pone.0052711 (PMC3527604; doi:10.1371/journal.pone.0052711)
Supplement: Table S3 — Primers used for qPCR analysis of the AR gene promoter region encompassing the three studied TBE sites. (DOC) [file pone.0052711.s006.doc]

**Supplementary Table S3.**

Primers used for qPCR analysis of the *AR* gene promoter region encompassing the three studied TBE sites.

TBE1 site sense

5’-CACCCGAGTAGCTGGGACTA-3’

TBE1 site antisense

5’-CAGTGGCTCATGCCTGTAATC-3’

TBE2 site sense

5’-TGCCAGTATGCCACCAGTAG-3’

TBE2 site antisense

5’-CACCATCTCACAGCCGATAA-3’

TBE3 site sense

5’- TGTTGGAAACATCAGGCAAA-3’

TBE3 site antisense

5’- CGTAAGGATTCGCTGAGAGG-3’
